# Prediction of neonatal acidosis using the cerebroplacental ratio at different gestational weeks: A case–control study

**DOI:** 10.1097/MD.0000000000016458

**Published:** 2019-07-19

**Authors:** Lin-Na Guo, Yi-Qing Chai, Shuang Guo, Zhi-Kun Zhang

**Affiliations:** Department of Ultrasound Centre, Tianjin Central Hospital of Obstetrics and Gynecology, Tianjin, China.

**Keywords:** acidosis, cerebroplacental ratio, doppler, neonate, ultrasonography

## Abstract

We evaluated the clinical value of the cerebroplacental ratio (CPR) in predicting neonatal acidosis according to the gestational weeks in late pregnancy.

From July 2016 to June 2017, 1018 neonates without acidosis and 218 neonates with acidosis (confirmed postpartum) underwent a prenatal examination and hospital delivery at 28 to 41^+6^ weeks in our hospital. The CPR was calculated as the ratio of the prenatal middle cerebral artery-pulsation index (MCA-PI) to the umbilical artery-pulsation index (UA-PI).

In neonates without acidosis, the fetal UA-PI decreased with increased gestational age during late pregnancy. Similarly, the MCA-PI decreased with increased gestational age, and decreased significantly during the full pregnancy term. Additionally, the CPR peaked in the middle of the late pregnancy period and then decreased. In contrast, in neonates with acidosis, the prenatal UA-PI increased significantly, MCA-PI declined significantly, and CPR declined significantly in relation to normal values (*P* < .05). For the prediction of neonatal acidosis, the UA-PI was suitable after 32 weeks and the MCA-PI was suitable before 37 weeks. The cutoff values of the CPR for the prediction of neonatal acidosis at 28 to 31^+6^ weeks, 32 to 36^+6^ weeks, and 37 to 41^+6^ weeks were 1.29, 1.36, and 1.22, respectively. Unlike the UA-PI and MCA-PI, the CPR was suitable as an independent predictor of neonatal acidosis at all late pregnancy weeks. In neonates with acidosis, the *z* score of the UA-PI increased significantly, whereas the *z* scores of the MCA-PI and CPR decreased significantly, in relation to normal values (*P* < .05).

The CPR can be used to evaluate the adverse status of fetuses during late pregnancy, providing an early prediction of neonatal acidosis.

## 1 Introduction

Population expansion is an issue in developing countries. Especially in areas with poor economic conditions, people need to consider the economic burden of bearing children, and high-quality fertility requirements have become a problem that cannot be ignored. Every family is particularly concerned about the health of the children; the relationship between doctors and patients has become tense and the contradictions are becoming more serious.

Neonatal acidosis suggests neonatal asphyxia, which can lead to the development of nervous system injuries, cerebral palsy, and mental retardation, and is a major cause of perinatal death. Its accurate prediction is undoubtedly important for prenatal management. At present, Doppler ultrasound is used to predict adverse pregnancy outcomes by detecting hemodynamic changes in the umbilical artery (UA), middle cerebral artery (MCA), renal artery, ductus venous, and internal umbilical vein.^[1]^ The fetal arterial blood flow resistance index (RI), pulsation index (PI), and peak systolic velocity to diastolic velocity ratio (S/D) reflect the peripheral circulation impedance and blood vessel perfusion.

Additionally, the cerebroplacental ratio (CPR), calculated as MCA-PI/ UA-PI, reflects fetal conditions and predicts newborn outcomes.^[2]^ However, controversy exists regarding whether the CPR can be used to routinely predict adverse pregnancy outcomes, what the cutoff value should be, and when it should be measured and applied for prediction.^[3]^ Furthermore, there are few studies regarding whether the CPR, measured during specific gestational weeks, can predict neonatal acidosis. Thus, we conducted a case–control study to observe changes in the CPR during the third trimester of pregnancy and investigate the clinical value of CPR in predicting neonatal acidosis. Specifically, we evaluated whether the CPR can be used as an independent risk factor for predicting neonatal acidosis at different gestational weeks, and determined initial estimates of the appropriate cutoff values and *z* scores.

## 2 Materials and methods

### 2.1 Patient selection

This case–control study utilized the results of umbilical artery blood gas analyses performed immediately after birth between July 2016 and June 2017 at the Tianjin Central Hospital of Obstetrics and Gynecology (China), which offers prenatal care and delivery services. Cases with umbilical artery blood at a pH ≥7.2 were allocated to the without acidosis group, whereas those with umbilical artery blood at a pH <7.2 were allocated to the acidosis group. We collected as many cases as possible to ensure the reliability of the results. To reduce the effects of confounding factors, the following exclusion criteria were applied: fetal heart disease, fetal malformations, chromosome abnormalities, maternal metabolic diseases, twin pregnancy, blood transfusion, cases of intrapartum abnormalities that may lead to acute neonatal asphyxia, and UA and MCA diastolic deletion cases with potentially severe intrauterine hypoxia. Finally, we selected 1018 neonates without acidosis (28–31^+6^ weeks, n = 328; 32–36^+6^ weeks, n = 360; 37–41^+6^ weeks, n = 330) and 218 neonates with acidosis (28–31^+6^ weeks, n = 47; 32–36^+6^ weeks, n = 82; 37–41^+6^ weeks, n = 89). None of the cases were repeated and we selected the results of ultrasound examinations performed within 1 day before birth. This study was approved by the local ethics committee, and informed consent was obtained from all patients for the purpose of publication.

### 2.2 Ultrasound procedure and parameters

A Doppler examination was performed using a Voluson E8 (GE Healthcare Austria Gmbh & Co OG, Tiefenbach 15, 4871 Zipf, Austria) ultrasonic machine equipped with a 2.0 to 5.0 MHz abdominal convex array probe. The fetal UA and MCA hemodynamic index was measured via color and spectral Doppler ultrasonography. The patient was placed in the supine position, with the head of the bed raised 30 degree, and probed without pressure. First, color Doppler was turned on and the sampling frame was adjusted. Then, spectral Doppler was turned on, and the sampling point was placed at the center of the vessel to orient the ultrasonic beam and blood flow in a parallel direction or at an angle of <30 degree. The sample volume was generally 2 to 3 mm, and we obtained >5 continuous blood flow spectra, which were automatically measured under blood flow parameters. The sampling point was then set at the umbilical cord, 2 cm from the placental attachment point, and the UA-PI was automatically measured. The PI was automatically measured from the middle segment of the MCA in the transthalamic plane. To limit experimental error, 3 measurements were taken and the highly repeated value was used. The CPR was then calculated as MCA-PI/UA-PI.

All of the participating researchers were experienced physicians who have been engaged in prenatal ultrasound diagnosis for many years. All researchers could skillfully operate the color Doppler ultrasound diagnostic instrument and had received standardized training.

### 2.3 Additional data collection

The following data were collected from detailed histories of the pregnant women: age, prepregnancy body mass index, menstrual history, pregnancy history, smoking and drinking history, amniotic fluid volume, delivery method, Apgar score 1 minute after birth, and the results of the umbilical artery blood gas analysis immediately after birth.

### 2.4 Statistical analysis

Continuous data are expressed as means ± standard deviation (SD). Categorical data are presented as numbers (percentage). Demographic characteristics were compared between the 2 groups using the independent samples *t* test and *χ*^2^ test, as appropriate. Changes in prenatal UA-PI, MCA-PI, and CPR across the 3 gestational weeks were evaluated using the F test, with least squares difference post hoc tests. Group differences in prenatal UA-PI, MCA-PI, and CPR according to gestational week were evaluated using the independent samples *t* test.

Logistic regression analysis was used to evaluate the predictive performance of prenatal UA-PI, MCA-PI, and CPR according to gestational week. Additionally, receiver-operating characteristic (ROC) curve analyses were performed, and cutoff values were determined. Additionally, a fractional polynomial regression analysis was performed. The statistical model described by DeVore^[4]^ was used to estimate *z* score reference intervals. The reference ranges for both mean and SD were constructed according to the gestational age as an independent variable, based on the best-fit models. *z* Scores were then calculated as follows: *z* score = (measured value – predicted mean)/predicted SD. The fractional polynomial regression analysis was performed using NCSS version 12.0 (NCSS, LLC., Kaysville, UT). All other analyses were performed using SPSS version 17.0 (SPSS, Inc, Chicago, IL). *P* < .05 was considered statistically significant.

## 3 Results

The acidosis group had higher gestational age (*P* < .001), higher prepregnancy body mass index (*P* = .008), lower amniotic fluid volume (*P* = .002), higher frequency of cesarean births (*P* < .001), and lower Apgar score 1 minute after birth compared to those in the without acidosis group. No other significant differences in background characteristics were observed between the 2 groups (Table 1).

**Table 1:**
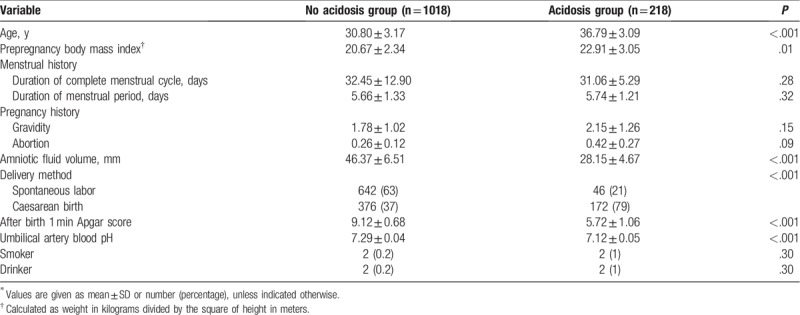
Demographic characteristics^∗^.

**Figure 1:**
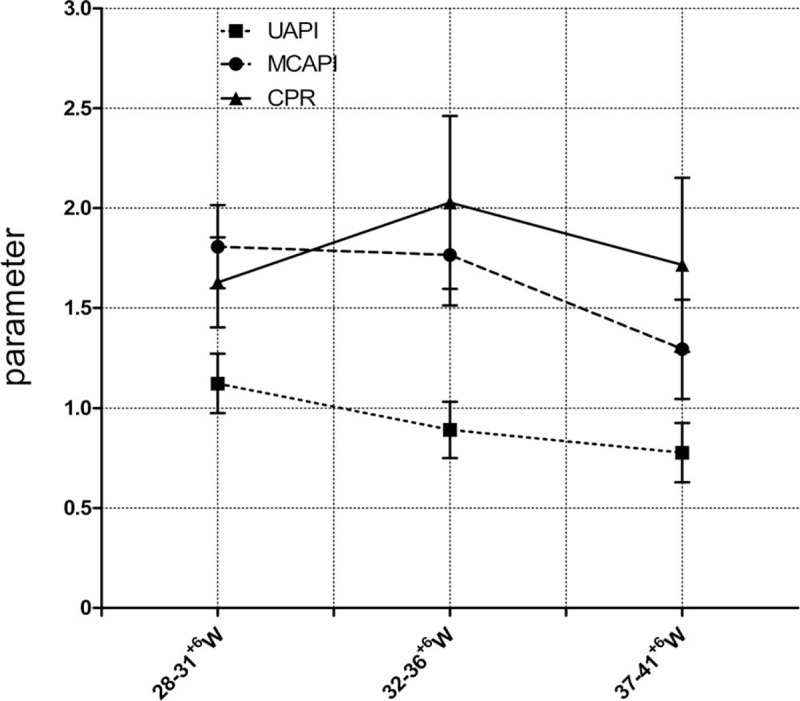
Changes of prenatal doppler parameters in neonates without acidosis at 3 weeks. The difference in UA-PI, MCA-PI, CPR between the 3 groups was found to be statistically significant, *P* < .001. Least squares difference post hoc tests were performed. The difference in UA-PI between the 3 groups was found to be statistically significant, *P* < .001. Between 28 to 31^+6^ and 32 to 36^+6^ weeks, between 28 to 31^+6^ and 37 to 41^+6^ weeks, between 32 to 36^+6^ and 37 to 41^+6^ weeks, the difference in MCA-PI was statistically significant (*P* = .02, *P* < .001, *P* < .001), the difference in the CPR was statistically significant (*P* < .001, *P* = .004, *P* < .001). CPR = cerebroplacental ratio, MCA-PI = middle cerebral artery-pulsation index, UA-PI = umbilical artery-pulsation index.

In neonates without acidosis, the prenatal UA-PI decreased with increased gestational age. Similarly, the MCA-PI decreased with increased gestational age, decreasing significantly over the full pregnancy term. The CPR peaked in the middle of the late pregnancy period and then decreased (Fig. 1). Differences in the UA-PI, MCA-PI, CPR among the 3 gestational subgroups were statistically significant (*P* < .001).

In neonates with acidosis, the prenatal UA-PI increased significantly, MCA-PI decreased significantly, and CPR decreased significantly at 3 weeks (*P* < .05) (Table 2).

**Table 2:**
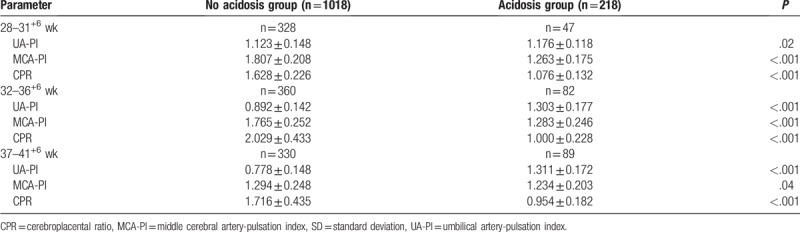
Changes of prenatal doppler parameters between the 2 groups at 3 weeks (mean ± SD).

After adjusting for the UA-PI and MCA-PI, only the CPR remained as an independent predictor of neonatal acidosis at 28 to 31^+6^ weeks (adjusted odds ratio [OR] 0.001; 95% confidence interval [CI], 0.000–0.298), 32 to 36^+6^ weeks (adjusted OR 0.184; 95% CI, 0.036–0.947), and 37 to 41^+6^ weeks (adjusted OR 0.139; 95% CI, 0.020–0.949) (Table 3).

**Table 3:**
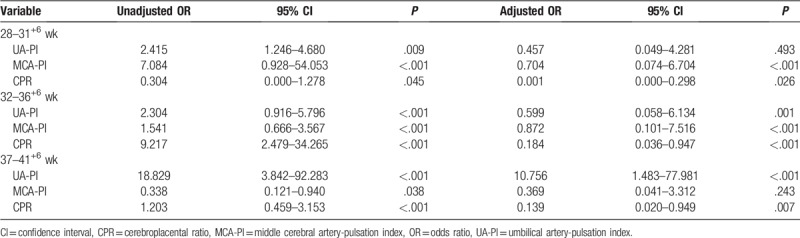
Logistic regression analysis to investigate whether prenatal doppler parameters are independent predictors of neonatal acidosis at 3 weeks.

**Figure 2:**
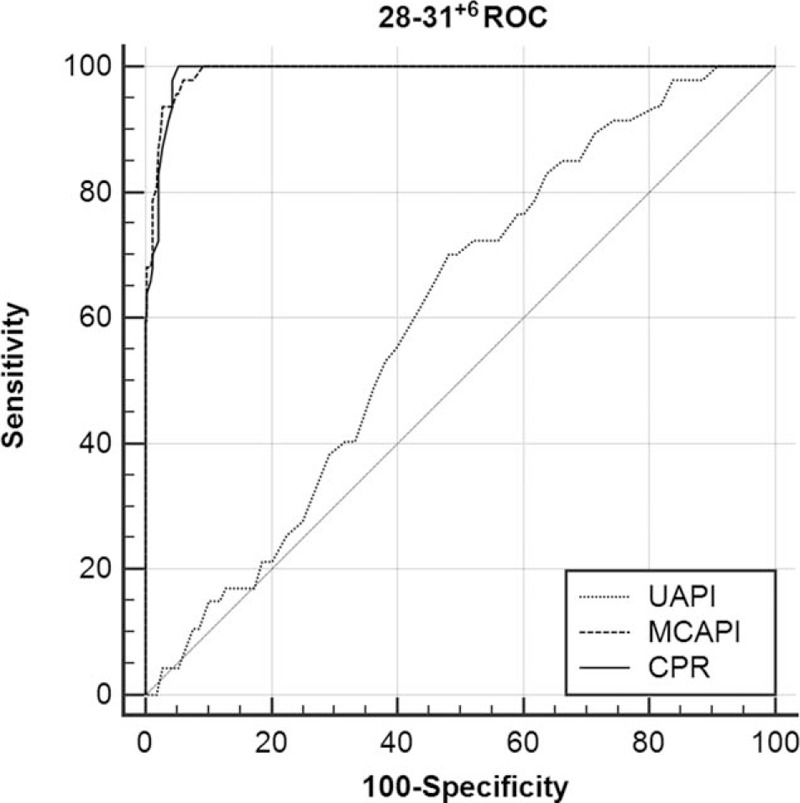
ROC curve analysis for the prediction of neonatal acidosis at 28 to 31^+6^ weeks (MCA-PI: AUC 0.991, 95% CI = 0.976–0.998, *P* < .001; UA-PI: AUC 0.606, 95% CI = 0.555–0.656, *P* = .02; CPR: AUC 0.991, 95% CI = 0.975–0.998, *P* < .001, respectively). AUC = the area under the curve, CI = confidence interval, CPR = cerebroplacental ratio, MCA-PI = middle cerebral artery-pulsation index, ROC = receiver-operating characteristic, UA-PI = umbilical artery-pulsation index.

**Figure 3:**
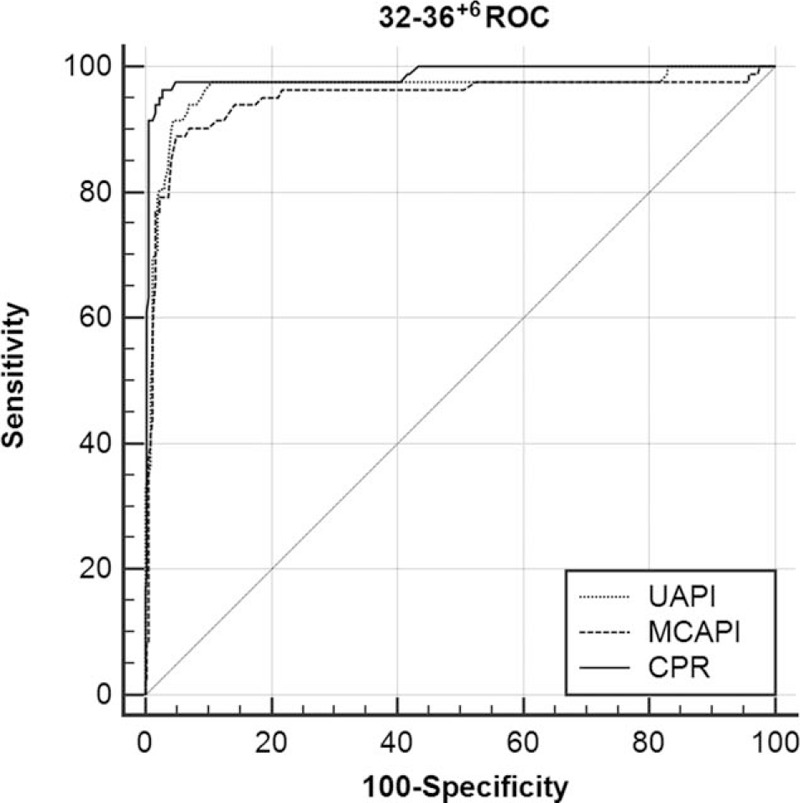
ROC curve analysis for the prediction of neonatal acidosis at 32 to 36^+6^ weeks (MCA-PI: AUC 0.949, 95% CI = 0.924–0.967, *P* < .001; UA-PI: AUC 0.965, 95% CI = 0.944–0.980, *P* < .001; CPR: AUC 0.985, 95% CI = 0.969–0.994, *P* < .001, respectively). AUC = the area under the curve, CI = confidence interval, CPR = cerebroplacental ratio, MCA-PI = middle cerebral artery-pulsation index, ROC = receiver-operating characteristic, UA-PI = umbilical artery-pulsation index.

**Figure 4:**
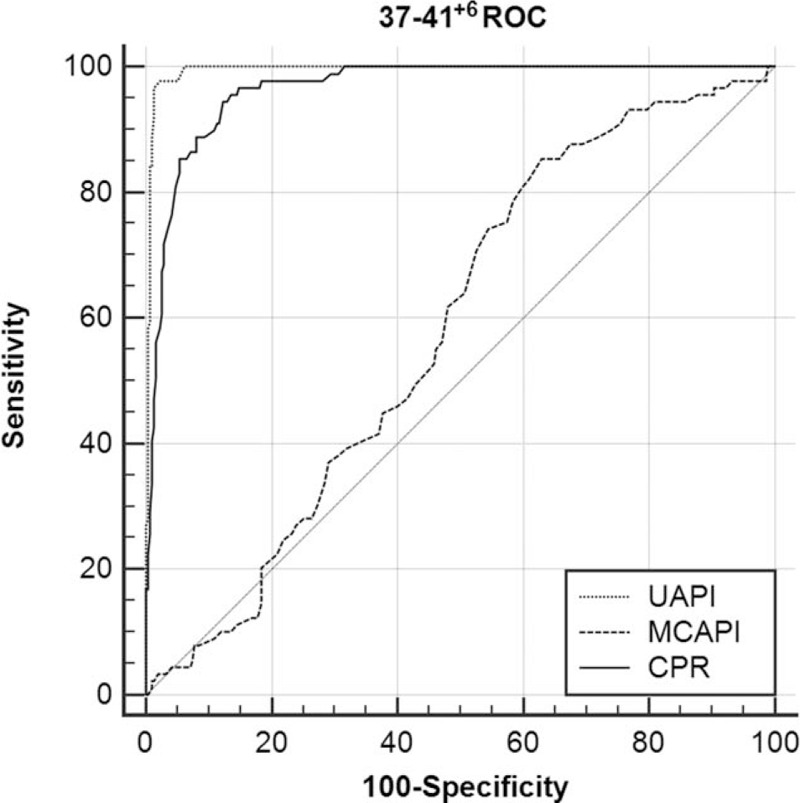
ROC curve analysis for the prediction of neonatal acidosis at 37 to 41^+6^ weeks (MCA-PI: AUC 0.582, 95% CI = 0.532–0.631, *P* = .04; UA-PI: AUC 0.994, 95% CI = 0.981–0.999, *P* < .001; CPR: AUC 0.965, 95% CI = 0.942–0.981, *P* < .001, respectively). AUC = the area under the curve, CI = confidence interval, CPR = cerebroplacental ratio, MCA-PI = middle cerebral artery-pulsation index, ROC = receiver-operating characteristic, UA-PI = umbilical artery-pulsation index.

The ROC curves for the UA-PI, MCA-PI, and CPR are shown according to gestational subgroups in Figures 2 to 4. The cutoff values of UA-PI for the prediction of neonatal acidosis at 28 to 31^+6^ weeks, 32 to 36^+6^ weeks, and 37 to 41^+6^ weeks were 1.12, 1.12, and 1.06, respectively. However, at 28 to 31^+6^ weeks, the adjusted OR was 0.457 (95% CI, 0.049–4.281) (*P* = .493), the ROC-the area under the curve (AUC) value reached 0.606, and the cutoff value was 1.12, which was equal to the normal mean value and did not denote predictive significance. Therefore, the UA-PI is only suitable for the prediction of neonatal acidosis after 32 weeks.

The cutoff values of the MCA-PI for the prediction of neonatal acidosis at 28 to 31^+6^ weeks, 32 to 36^+6^ weeks, and 37 to 41^+6^ weeks were 1.52, 1.44, and 1.37, respectively. However, at 37 to 41^+6^ weeks, adjusted OR was 0.369 (95% CI, 0.041–3.312) (*P* = .243), the ROC-AUC value reached 0.582, and the cutoff value was 1.37, which was higher than the normal mean value and did not denote predictive significance. Therefore, the MCA-PI is only suitable for the prediction of neonatal acidosis before 37 weeks.

The cutoff values of the CPR for the prediction of neonatal acidosis at 28 to 31^+6^ weeks, 32 to 36^+6^ weeks, and 37 to 41^+6^ weeks were 1.29, 1.36, and 1.22, respectively. The CPR generated high ROC-AUC values with considerable stability. Thus, the CPR can be used as an independent predictor of neonatal acidosis in the third trimester.

**Figure 5:**
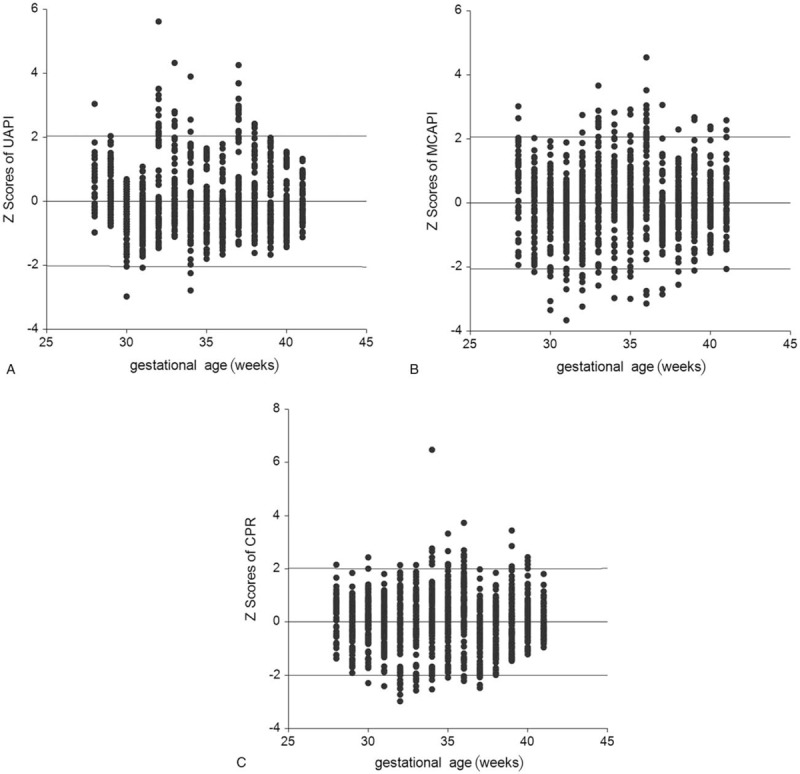
Scatterplots of *z* scores for UA-PI (A), MCA-PI (B), and CPR (C), as a function of gestational age, indicating adequate model fit. CPR = cerebroplacental ratio, MCA-PI = middle cerebral artery-pulsation index, UA-PI = umbilical artery-pulsation index.

The predicted means and SD of the UA-PI, MCA-PI, and CPR (as a function of gestational age) in the best-fitting regression models are presented in Table 4. In neonates with acidosis, the *z* score of the UA-PI increased significantly, whereas the *z* scores of the MCA-PI and CPR decreased significantly, in relation to normal values (*P* < .05) (Table 5). The *z* scores were symmetrically distributed above and below zero across the entire gestational age range. The number of *z* scores outside of the range ± 2 SD did not exceed 10%, as expected (Fig. 5).

**Table 4:**
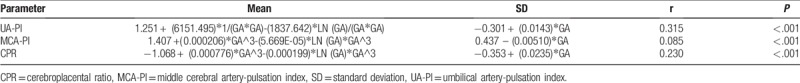
Regression models for predicted mean and SD of prenatal doppler parameters based on gestational age.

**Table 5:**
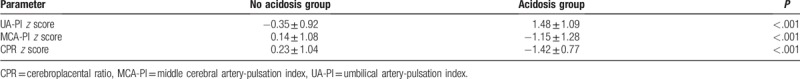
*z* Scores of prenatal doppler parameters between the 2 groups (mean ± quartile).

## 4 Discussion

The present study found that, in neonates without acidosis, CPR values peaked in the middle of the late pregnancy period (at 32–36^+6^ weeks) and then decreased, which is consistent with the study by Morales.^[5]^ This pattern of results was because of significant changes in the UA-PI and MCA-PI among the 3 gestational subgroups. In contrast, the prenatal CPR values in the acidosis group decreased significantly in relation to normal values. When fetal intrauterine hypoxia occurs, the blood flow redistributes, increasing the UA blood flow index and decreasing the MCA blood flow index; thus, a decrease in the CPR during late pregnancy, as observed in the neonates with acidosis, denotes the presence of brain protecting effects.^[6,7]^ Furthermore, based on the adjusted ORs, the present study found that the CPR, but not the UA-PI or MCA-PI, could be used as an independent predictor of acidosis during all third-trimester gestational weeks.

In the present study, the fetal UA-PI in neonates without acidosis significantly changed during late pregnancy, peaking at 28 to 31^+6^ gestational weeks, decreasing at 32 to 36^+6^ weeks, and decreasing to its lowest value at 37 to 41^+6^ weeks, consistent with the study by Thuring et al.^[8]^ To ensure the blood supply of the fetus, the UA blood vessels expand and the flow rate increases, resulting in the observed changes in the UA-PI. When fetal intrauterine hypoxia occurs, fetal-placental circulation resistance increases and the hemodynamic indices of the UA increase.^[9]^ Although prenatal UA-PI increased significantly in the acidosis group, the logistic regression analysis showed that the prediction of neonatal acidosis by the UA-PI was only suitable after 32 weeks.

The prenatal MCA-PI in neonates without acidosis peaked at 28 to 31^+6^ weeks in the present study. At this time, the brain is not well developed, the blood vessels are slender, and the resistance is high. To ensure brain development, the MCA-PI decreases significantly at 37 to 41^+6^ weeks,^[10,11]^ as observed in the neonates without acidosis in the present study. When fetal hypoxia occurs, blood flow is redistributed to ensure the brain's blood supply (the “brain protective effect”).^[12]^ Although the prenatal MCA-PI decreased significantly in the acidosis group, the logistic regression analysis showed that the prediction of neonatal acidosis by the MCA-PI was only suitable before 37 weeks. Consistent with this, Morales et al^[13]^ also suggested that a reduction in the MCA-PI predicted adverse fetal pregnancy outcomes at 28 to 32 weeks, but not after 34 weeks.

Adamson et al^[14]^ first proposed perfusion impedance theory. The ratio of the blood flow parameters (MCA-PI/UA-PI) is used to eliminate interfering factors present in both measures, and better reflects the blood flow distribution in the whole fetal body than that in a single vessel. In the early stages of hypoxia, the CPR can reflect the systemic fetal blood flow distribution, and the prediction of adverse pregnancy outcomes is, in turn, more sensitive.^[15]^ The PI is the ratio of the fluctuating amplitude to the mean blood flow velocity; it is highly stable and reflects the blood conditions of the entire heart cycle.^[16]^

The meaning and cutoff values for the CPR broadly differ between studies. Karlsen et al^[17]^ suggested that MCA-PIs <5% and CPRs <10% are closely related to adverse perinatal pregnancy outcomes and that the CPR has a better prediction performance. Maged et al^[18]^ suggested that the likelihood of poor pregnancy outcomes increases for CPRs <1.05. Khalil et al^[19]^ showed that CPRs <0.6765 predict fetal death and neonatal death. Other researchers have argued that the CPR cannot routinely predict adverse outcomes in late pregnancy and that it only appears to be effective when applied to high-risk populations.^[20,21]^ Decreased CPR suggests poor placental perfusion and injury.^[22,23]^ Furthermore, the present study result suggest that the CPR can be applied as an independent routine prognosis predictor in late pregnancy.

The present study, to the best of our knowledge, is the first to establish *z* score models for the UA-PI, MCA-PI, and CPR based on the gestational age using fractional polynomial regression analysis. Additionally, we evaluated a relatively large sample, ranging in age from the 28^th^ week of pregnancy to term. Furthermore, the *z* scores were carefully examined to confirm normality and we also performed a regression analysis of the SD separately, which is essential for *z* score assessment. Additionally, the *z* score distribution against gestational age was tested for an adequate model fit. The *z* score is more informative than the cutoff value for indicating an abnormality, as it can accurately quantify the degree of deviation from normal, and it can be used for diagnosis and monitoring, thus providing more clinical application options.

It should be noted that many factors can interfere with the measurement of the UA-PI and MCA-PI, which had large variabilities. As the CPR is the ratio of the MCA-PI to the UA-PI, accurate measurements of these blood flow indices are vital for minimizing measurement error. Second, in accordance with known changes in the UA and MCA over the gestational weeks in late pregnancy, and to facilitate diagnosis, the CPR was analyzed by 28 to 31^+6^-, 32 to 36^+6^- and 37 to 41^+6^-week gestational age subgroups. When severe fetal intrauterine hypoxia occurs, the UA diastolic blood flow disappears and even reverses, and the MCA index has biphasic changes, declining to its lowest point and then increasing. Thus, the ratio between these indices cannot be used to evaluate the hypoxia state; although the CPR can be used to evaluate early intrauterine hypoxia conditions, it cannot be used to evaluate decompensation conditions in severe cases of fetal hypoxia.

The present study has several limitations. The present study excluded pregnant women with comorbidities; thus, the CPR values of such patients require further research. As pregnancy age, body mass index, and population composition could have impacted the statistical results, larger logistic regression analysis models are needed. The diagnostic accuracy of the CPR cutoff values also requires further investigation with greater data support. Finally, prospective serial studies are needed to verify the validity of the *z* scores.

The CPR eliminates interfering factors, better reflects the blood flow distribution in the whole body than that in a single vessel, and can be used to evaluate fetal intrauterine hypoxia earlier, with better prediction of neonatal acidosis, than can other indices. Accurate prediction of neonatal acidosis, with effective recommendations to pregnant women and their families are important for prenatal management. The present study results suggest that the CPR is worthy of clinical application.

## Author contributions

**Conceptualization:** Lin-Na Guo, Zhi-Kun Zhang.

**Data curation:** Lin-Na Guo, Yi-Qing Chai, Shuang Guo.

**Formal analysis:** Lin-Na Guo, Yi-Qing Chai, Shuang Guo, Zhi-Kun Zhang.

**Funding acquisition:** Lin-Na Guo, Zhi-Kun Zhang.

**Investigation:** Lin-Na Guo, Yi-Qing Chai, Shuang Guo, Zhi-Kun Zhang.

**Methodology:** Lin-Na Guo, Yi-Qing Chai, Shuang Guo, Zhi-Kun Zhang.

**Project administration:** Lin-Na Guo, Zhi-Kun Zhang.

**Resources:** Yi-Qing Chai, Shuang Guo, Zhi-Kun Zhang.

**Software:** Shuang Guo.

**Supervision:** Lin-Na Guo, Zhi-Kun Zhang.

**Validation:** Lin-Na Guo, Zhi-Kun Zhang.

**Writing – original draft:** Lin-Na Guo.

**Writing – review & editing:** Lin-Na Guo, Zhi-Kun Zhang.

Lin-Na Guo orcid: 0000-0003-4583-1345.
